# Landmark-Based Features for Vehicle Trajectory Anomaly Detection from Traffic Video in Urban Intersections—A Case Study

**DOI:** 10.3390/s26103027

**Published:** 2026-05-11

**Authors:** Nicolae Cleju, Constantin Catargiu

**Affiliations:** Faculty of Electronics, Telecommunications and Information Technology, Gheorghe Asachi Technical University of Iasi, 700506 Iași, Romania; constantin.catargiu@student.tuiasi.ro

**Keywords:** anomaly detection, trajectory, features, landmarks

## Abstract

We study trajectory feature representations in the context of detecting spatially anomalous vehicle trajectories in urban intersections, using trajectory data from video streams captured by camera monitoring systems. These trajectories are extracted using an object detection pipeline and have particular characteristics like short lengths, variable endpoints, and other viewpoint-dependent detection artifacts, which make existing spatial feature approaches less effective. We introduce two feature representations adapted for intersection-level trajectories, based on distances to a fixed set of landmark points, which provide fixed-length vectors compatible with common tabular anomaly detector algorithms. We evaluate using a dataset of 5378 labeled trajectories collected from camera recordings in one deployment site, as well as on other existing city-wide benchmark datasets, showing that, in the evaluated setting, the proposed feature representations improve upon several existing spatial features and enable better detection of both shape and placement anomalies.

## 1. Introduction

Trajectory data are ubiquitous in today’s scientific literature, thanks to the widespread proliferation of location-based tracking devices and sensors. Whether they are about human mobility, vehicle traffic patterns, animal behavior, or even movements of meteorological phenomena like hurricanes, trajectories are a rich source of information, leading to a vast body of literature on analysis, classification, and other data-mining techniques tailored for them.

Analysis of traffic-based trajectories is a particularly important aspect within the emerging concept of smart cities since traffic management is an important part of daily urban life. While most of the existing literature focuses on long, city-wide trajectories recorded with GPS and other location-based sensors, the availability of video-based traffic monitoring systems provides an alternative source of relevant trajectory data, particularly suited for busy urban hubs and intersections, which are likely to be already equipped with such video monitoring systems. The use of AI-based object detection can help with automatic extraction and analysis of trajectories from traffic monitoring streams, providing a completely remote and non-intrusive solution for trajectory analysis, without needing to access the location data from the vehicles themselves.

Anomaly detection is an important aspect in trajectory analysis, helping identify problematic and risky traffic patterns. In general, traffic anomalies include spatial, temporal (speed-related), or more complex behavioral aspects (e.g., aggressive driving). In this paper we consider only spatial anomalies, i.e., trajectories with abnormal shape or placement. We study spatial feature representations for a specific type of trajectory, namely vehicle trajectories in urban intersections extracted from traffic video using AI-based object detection and tracking, in the context of anomaly detection tasks. We investigate whether spatial trajectory features designed for general city-wide trajectories are suitable for intersection-level trajectories or whether these can be better handled with more specific features. We introduce two novel spatial feature representations for such trajectories, based on computing distances to a fixed set of reference points (landmarks), and we evaluate them using data collected from a busy intersection in Iași, Romania, consisting of 5378 vehicle trajectories from a 3 h long video extracted using the AI-based pipeline and hand-labeled for anomalies in shape and placement. Our analysis shows that they provide better results than several existing spatial feature approaches, especially for intersection-level trajectories, in the context of our preprocessing pipeline. We also discuss the importance of preprocessing in the context of trajectory data extracted from video streams with AI-based tools.

Our work follows the classical pattern of feature extraction followed separately by outlier detection. This allows us to focus on evaluating the features independently of the detection model. Furthermore, the feature representation could also be updated to match specific characteristics of intersections monitored.

The major contributions of this paper are the following:

- We introduce two types of spatial trajectory features for anomaly detection tasks, based on distances to a fixed set of reference points.
- We evaluate them in the context of a target urban intersection, using a new labeled dataset with 5378 intersection-level vehicle trajectories, extracted from traffic video with AI tools, followed by a specific preprocessing pipeline.
- We show that the proposed representations yield better results than several existing spatial feature approaches for trajectories in the evaluation site when coupled with our preprocessing pipeline. We also discuss relevant factors such as landmark placement and provide complementary results on existing city-wide trajectory datasets, which suggest feature versatility in different contexts.

We evaluate the proposed features with intersection trajectory data collected from a single intersection site, where our traffic monitoring pipeline is set up; in general, intersection-level trajectory datasets are scarcely available. As such, the work should be regarded as a feature-analysis case study targeting a localized intersection setup, motivating a deeper look into spatial feature representations adequate to intersection-level trajectories extracted from video data, rather than a full-scale anomaly detection solution.

The rest of the paper is organized as follows. In Section 1, we review the most relevant literature related to our work. In Section 2, we define the problem, discuss some limitations of existing approaches, and introduce the proposed landmark-based methods for trajectory features, as well as the new dataset of intersection-level trajectories. Several anomaly detection results are presented in Section 3, followed by a discussion in Section 4. Finally, we conclude the paper in Section 5.

### Related Work

There is a vast body of literature on trajectory analysis and, specifically, anomaly detection. Several review papers [1,2] summarize the prevailing approaches in the field, as well as open challenges. We summarize below the major directions in the literature, focusing on anomaly detection approaches, types of trajectory data, and acquisition methods.

When it comes to the approach used for anomaly characterization and detection, there are several major directions in the literature. One traditional approach is to characterize trajectories using features extracted from their shape or location and then use standard vector-based outlier detection algorithms. For example, Rintoul and Wilson [3] use 12 such features, mostly describing the geometrical shape pattern, in order to implement data mining techniques with the goal of retrieving trajectories with similar patterns. Features used include length, various measures of curvature, and descriptors of the convex hull encompassing the trajectory. In [4], a histogram-like feature vector is proposed, which describes the circular distribution of trajectory points around the trajectory centroid. In [5,6], a trajectory is characterized simply by computing the shortest distances from the trajectory to a set of external reference points (landmarks), thus forming a feature vector of distances; several types of distances are proposed and used. We discuss both these algorithms in more detail in Section 2.

Note, however, that the selection of optimal features is highly problem-specific and sensitive to changing conditions and different types of trajectory data included. For example, an in-depth analysis and comparison of geometric and kinematic descriptors in [7], albeit done in the context of maritime ship classification from trajectories, reveals that the predictive power of models depends significantly on the ship classes included in the dataset, without a clear and consistent set of optimal features. In [8], we find another in-depth comparison of several shape-describing trajectory features, evaluated in a clustering problem.

Instead of analyzing trajectories indirectly through features, another classic approach relies on defining custom distance metrics, specifically designed for trajectory data, which can quantify into a single number the difference (or the similarity) between two trajectories. Classic distance measures include the Hausdorff, Frechet, and edit-based distances, together with a multitude of variants, as well as more recent variants such as SSPD. A review of commonly used measures is available in [9]. These distances can be applied to sub-trajectories as well. A classic algorithm, TRAOD [10], splits trajectories into sub-trajectories, approximates them with segments, and relies on specific segment-based distance measures to identify anomalous sub-trajectories. Despite being easy to interpret, trajectory distances have some significant disadvantages. First, they cannot be used with a vast majority of vector-based algorithms, which require vector-space operations (e.g., same dimensions, means, projections, Euclidean distances), whereas trajectory distances provide only distance values between pairs of trajectories, without a vector-space embedding. Second, since trajectory distances need to be computed between every pair of trajectories separately, they are not a good fit for continuous operation when new trajectory data is continuously incoming over a long time. As such, trajectory distances tend to work well only with certain classes of algorithms, like density-based, agglomerative, or affinity propagation [9], but not with others.

Another major approach relies on converting trajectories into a grid-based representation. Mapping them over a fixed grid has the advantage of producing a fixed-size representation, which can then be used more easily with common vector-based algorithms. Grid-cell representations are used in classic algorithms like iBAT [11], which relies on isolation forest-based detection over the grid support, and iBOAT [12], which further proposes an online solution that allows evaluating trajectories on the fly as new points are added. The mapping of trajectories over a grid can be done with various degrees of refinement, including eliminating outlier “abnormal” grid cells, as in [13].

Another vast emerging approach is based on representation learning, which relies on learning trajectory embeddings in a latent feature space, generally using neural network architectures. In [14], recurrent neural networks (RNNs) are used, specifically LSTM and GRU architectures; RNNs have the advantage of accepting variable-length trajectories as input, encoding them into fixed-length vectors. Variational autoencoders (VAEs) are used in [15], operating both on raw trajectory GPS coordinates and on externally available route features, to detect abnormal trajectories in a real-time setting. VAEs are also used in [16], which learns a probabilistic metric rule to differentiate normal from abnormal trajectories even for unseen types of anomalies. In [17], a lightweight CNN-based model is trained on traffic trajectories, which are first converted into a rectangular grid-based representation; however, their aim is trajectory prediction and not anomaly detection. In [18], the grid-based and road-based representations of a trajectory are fused into a single representative vector, using CNN-based and GraphNN-based encoders followed by a cross-attention fusion module.

Transformer architectures have become popular in recent years for learning trajectory embeddings, exploiting the fact that trajectories are sequences of points or locations. Mbuya et al. [19] propose LM-TAD, a transformer-based autoregressive model trained to learn the distribution of points given a preceding window, relying on the perplexity metric to identify anomalies. Their framework allows more general definitions of a trajectory, not merely as a sequence of GPS points but also as a sequence of stay points (“home”, “office”, “restaurant”) or even as a sequence of activities. As a downside, when using GPS points, they need to be discretized into a spatial grid in order to have a discrete set of elements. (e.g., a cell size of 100×100 m for the Porto dataset). In [20], two types of embeddings are extracted from trajectories and then fed into a parallel LSTM-based RNN architecture for anomaly detection. Lohrer et al. [21] propose a deep BERT-based transformer encoder model architecture. In their approach, anomaly detection is formulated as a group anomaly detection problem, by considering the trajectory as a group of sequential segments. Li at al. [22] take a denoising-based approach for anomaly detection; they propose a diffusion-based denoising model relying on temporal and spatial transformer-based embeddings to learn the denoising process for noisy trajectories and then use the reconstruction error as a metric for detecting anomalous trajectories. Hou et al. [23] show that a transformer-based VAE outperforms conventional and LSTM-based VAEs in the context of identifying anomalous ship trajectories. Ship trajectory anomalies are also the use case in [24], which uses a transformer based on ProbSparse attention to predict the next ship position, detecting an outlier when it deviates too much. A recent review of deep learning approaches in trajectory analysis is available in [25].

Most of the literature uses GPS-based trajectory data, thanks to the availability of large datasets [26,27,28,29]. The trajectories are considered sparse, as the sampling rate is usually of the order of one sample every few seconds, or even tens of seconds. For vehicles, many trajectories are collected from city-wide taxi trips. The use of video-based trajectory data is much less common but has increased with the popularity of AI-based object detection. As early as 2006, Atev et al. [30] introduced clustering of intersection trajectories, extracted from video data with a custom region-based vehicle tracker. More recently, Riehl et al. [31] investigate trajectory extraction from aerial footage, using a variety of object detection models and trajectory post-processing (e.g., smoothing). A complete pipeline for trajectory extraction and integration from multiview traffic video in an intersection is presented in [32]; however, no code is provided for reproducibility purposes. In [33], another complete pipeline for trajectory extraction from aerial footage is presented, and the dataset and code are fully provided; while it is specifically designed for drone-based bird’s-eye view video (e.g., it includes video stabilization), it may also be adapted to traffic camera videos.

The solution proposed in [34] has the same goal as our work, namely, unsupervised anomaly detection of vehicle trajectories in intersections, but it uses a completely different approach. Trajectories are determined using YOLOv8 object detection and tracking and then are translated into a patch-based map representation of the intersection, the trajectory being defined as a sequence of patches traveled by the vehicle. Anomaly detection is achieved by learning a behavioral model of trajectories, i.e., what types of objects are typically found in each patch, how much time is spent in each patch, and which patches are visited next, and identifying which trajectories deviate from the learned models. Unfortunately, a direct comparison is not possible, since no public implementation is available and the results are evaluated on a private dataset. This highlights the need for open datasets and benchmarks.

Summarizing, urban trajectories extracted from video are a relatively under-explored subset of trajectory data, with the potential to become increasingly important in the future. Given that optimal feature selection is highly problem- and data-specific, it is not clear whether existing anomaly detection approaches developed for sparse, long GPS-based vehicle trajectories are also effective for this kind of dense, short, intersection-level trajectories extracted using AI. We address this question in the current work.

## 2. Methods

### 2.1. Background

A trajectory *T* is typically defined as a sequence of timestamped points:

(1) $$T=\{\left(p_{1},t_{1}\right),\left(p_{2},t_{2}\right),\ldots ,\left(p_{n},t_{n}\right)\},$$

where $p_{i}=\left(x_{i},y_{i}\right)$ are coordinates in a certain system, and $t_{i}$ is the corresponding moment in time. Following the commonly used definition of outliers in literature [35], we define a trajectory anomaly (or outlier) as a trajectory that deviates so much from the rest of the dataset as to raise suspicion of a different, out-of-the-ordinary movement behavior. An unusual movement pattern may indicate a possible risk or traffic incident and warrant further inspection, possibly by a human operator. We use the terms “anomaly” and “outlier” interchangeably.

In this paper, we consider only the spatial component of the trajectories, i.e., we disregard the timestamps, because we are interested primarily in trajectories of vehicles crossing an intersection, which are short in both length and duration. For these trajectories, the duration of the trajectory is usually small, and the anomalies tend to reflect mostly in the geometrical shape or placement. For example, in the dataset we have collected for this paper, after preprocessing, the trajectory durations are between 1.3 and 10 s.

The focus in this work is on trajectories extracted from video streams using AI object detection and tracking models, specifically in intersection areas, which are a prime focus for smart city initiatives. Compared with existing approaches in the literature, vehicle trajectories in intersections, extracted from video monitoring systems, have some special characteristics:

- The trajectories are short, ranging from a few meters to a couple of tens of meters, depending on the field of view of the video camera. For example, in the dataset introduced in this paper, the lengths are from 5.17 to 44.63 m.
- The trajectories are sampled with a very high rate (e.g., 20 Hz), corresponding to the frames-per-second parameter of the video source.
- Vehicle trajectories as they cross the intersection are characterized by smooth curves rather than sharp turns due to the fact that vehicles have limited maneuverability over short distances, coupled with the high sampling rate of the video monitoring system. Also, the trajectories are not restricted to a rigid grid, as in the case of long-distance trajectories, which are mapped over existing city street grids (see, for example, the Beijing taxi T-Drive datasets [26,27]).
- Trajectories can be anomalous either in localization or in shape. An overly sinuous trajectory may be anomalous due to its uncommon shape, but even a geometrically regular trajectory may be anomalous simply because it is shifted a few meters from the usual lane where it is performed.

The fact that the trajectories are extracted from video streams with AI tools, rather than using GPS sensors mounted on the vehicles themselves, leads to several other characteristics:

- There is significant variability in the location of the trajectory endpoints, even for vehicles that travel along the same route. Since certain vehicles may be detected by the AI model with higher confidence than others as they enter the field of view, the starting point of the recorded trajectories may differ, even for vehicles that enter via the same lane. This is also true for the end of a trajectory, especially when the objects are traveling away from the camera into the distance: as the vehicles become smaller, they are eventually lost by the object detection and tracking, but some vehicles are detected far longer than others.
- There is also high variability in terms of initial movement state at the beginning of the trajectory. Some vehicles may be detected as they are waiting at a traffic light, and therefore, their recorded trajectory starts with a stationary part with an unpredictable duration. Other vehicles enter the field of view while they are already in motion, so their trajectories start abruptly with a higher speed. The trajectories being short, the impact of this variability is not negligible.
- Object detection from video produces noisy trajectories, with possible gaps caused by occlusions or missed detections. Certain preprocessing operations, e.g., smoothing and interpolation, are commonly used to handle these issues [36] before the trajectories are ready to be used in algorithms.

Our aim is to design anomaly detection solutions that are robust to this variability, induced by the nature of trajectories in intersections and by the limitations of the video-based detection methods.

### 2.2. Relevant Existing Anomaly Detection Approaches

We first provide the technical details of the two anomaly detection approaches most relevant to our work as the basis for our proposed methods.

#### 2.2.1. MiPo Features and Tabular Outlier Detectors

MiPo [4] is a feature extraction method for spatial trajectory anomaly detection based on characterizing the angular distribution of the trajectory points around a reference point, taken as the middle point between the trajectory endpoints. It produces a fixed-length feature vector, irrespective of the trajectory length, which can then be used with standard tabular-based outlier detection algorithms. In brief, the method consists of the following steps:

1. The line connecting the two trajectory endpoints is taken as the reference axis, and the middle point between the endpoints is the reference point.
2. For each point on the trajectory, compute the polar coordinates with respect to the reference point and axis, i.e., compute the distance to the reference point and the angle of the connecting segment with respect to the reference axis.
3. Group the angles in *n* bins, spanning the whole [0,2π] range, and for each bin, record the average distance of the points (distance features) and the number of points in that bin (point features). Several practical optimizations are also suggested, such as special handling of empty bins, normalizing the distances, or using a different number of bins for the distance and point features.

The result is a histogram-like set of values describing the count and the average distance of the trajectory points, clockwise around the reference point and axis, using angle binning. Thus, each trajectory is represented by a fixed-size feature vector, which makes the method appropriate to use with standard tabular-based outlier detection algorithms (the authors use LOF [37]). The size of the feature vector is $n_{d}+n_{p}$, where $n_{d}$ is the number of bins used for distance values, and $n_{p}$ is the number of bins for point counts.

The MiPo method is closely related to the shape context introduced in [38], which characterizes a shape via a 2D, rotation-invariant log-polar histogram of shape points around a reference point.

The experiments in [4] are done on long city-wide trajectories and indicate good performance compared with other methods from the literature, including classic algorithms like iBAT [11] and iBOAT [12] and newer solutions like [39]. However, several possible limitations of the method are apparent:

- The MiPo method is inherently rotation and translation invariant because it relies on the position of points only with respect to the reference middle point and axis, which are both defined only by the endpoints. Any translation and/or rotation does not change the distances and angles. Thus, it is appropriate for capturing anomalies in shape but not in absolute placement, which is required in intersection-level trajectories.
- The angular coordinates of the points become less reliable for points that are close to the reference point, where position noise can lead to large changes in angle. This may happen for trajectories which are short and/or mostly linear, that are common in intersection-level trajectories, including in our dataset.

To address these limitations, we adapt the MiPo method to work with a fixed set of reference points in Section 2.3.2.

#### 2.2.2. Landmark-Based Distance Features

Another recent method for trajectory feature extraction is proposed in [5,6]. The authors characterize a trajectory only by computing the shortest distances from a set of fixed landmarks to that trajectory. This produces a fixed-length feature vector, which, similar to MiPo, can be used with standard vector-based algorithms.

Several types of distance measures for computing these feature vectors are proposed:

- Unsigned distances [5]: Take the shortest distance from the landmarks to any segment in the trajectory. The feature vector consists of these distance values in a fixed order. This is the simplest approach but has the drawback of being insensitive to direction: if the trajectory is traveled in the opposite direction, the distances remain the same, and thus the feature vector is identical.
- Projected points [5]: Take the projection points of these landmarks onto the trajectory and assemble the feature vector out of the coordinates of these points. Similar to the previous method, this is also insensitive to direction.
- Signed distances, introduced in [6]: Compute a signed distance from the landmarks to the trajectory, weighted by a Gaussian damping factor that depends on the distance to the trajectory. The feature vector consists of the signed distances from the landmarks to the trajectory.

These types of feature vectors are computationally simple and are shown by the authors to have good performance in trajectory classification using common algorithms such as KNN and SVM.

### 2.3. Landmark-Based Trajectory Features

Starting from the two existing methods described above, we now introduce our proposed methods for trajectory feature extraction and outlier detection, focusing on intersection-level trajectories. We refer to them together as landmark-based methods, since they rely on a set of reference points (landmarks) external to the trajectories themselves.

#### 2.3.1. Landmark Distances (LDF)

We propose to use the distance features introduced in [5] to characterize trajectories, using a fixed set of *N* reference points scattered across the intersection. For each reference point, we compute the shortest distance to the trajectory, resulting in *N* features per trajectory. We use both the signed and unsigned distances introduced in [6]. The distances are point-to-segment, i.e., the perpendicular projections from the reference points onto the trajectory can land anywhere along the segments connecting two consecutive trajectory points. Throughout the rest of the paper, we refer to this approach as landmark distance features (LDF). The dimension of the feature vector is equal to *N*, the number of reference points chosen.

**Figure 1:**
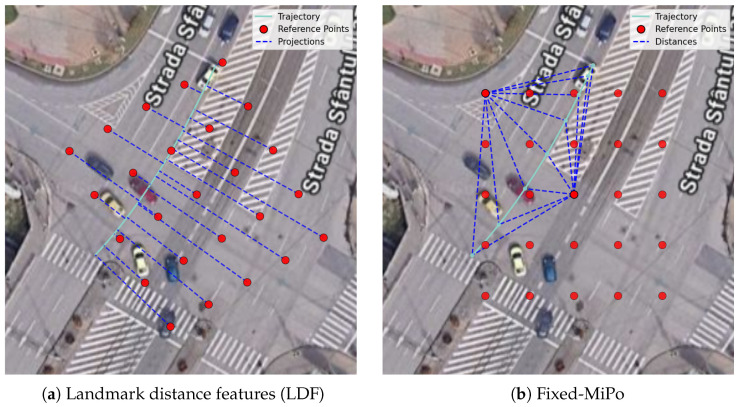
Illustration of the trajectory feature extraction methods based on a grid of fixed landmarks (possibly rotated). (**a**) Landmark distance features (LDF): shortest distances from the reference points to the trajectory, used to characterize the trajectory. The distances can be signed or unsigned. (**b**) Fixed-MiPo: segments from the trajectory points to two reference points, which are then binned by angle to form MiPo-like features.

The method is illustrated in Figure 1a, which shows the shortest distances from a set of reference points arranged in a grid to a sample trajectory.

The reference points can be arranged in various ways, which are explored in the experiments. The basic arrangement is a rectangular grid of points, possibly rotated at various angles (e.g., to align with the main street directions), as visible in Figure 1. Optionally, a controlled amount of randomness may be added to their locations. Another option is to select random points uniformly within the intersection area.

#### 2.3.2. Fixed-MiPo

A second proposed method is a modification of the MiPo features [4], aiming to exploit their efficiency, but without the rotation and translation invariance, which are not desired in our case.

We propose to compute the MiPo features with respect to a fixed set of reference points scattered across the intersection and then perform the angle binning with respect to a fixed north–south axis. In other words, instead of computing the polar coordinates of the trajectory points with respect to the middle point and axis defined by the trajectory endpoints, as in normal MiPo, we compute them with respect to a fixed reference point and the geographical north–south axis. We repeat this procedure for each reference point and concatenate the resulting features.

We refer to this method as Fixed-MiPo, and it is summarized in Algorithm 1. Figure 1b illustrates the method for a sample trajectory, showing the segments connecting the trajectory points to two reference points, situated on opposite sides of the trajectory. For each reference point, we bin these segments by angle with respect to the north–south axis and record the average distance and the number of segments per angle bin, as in MiPo. This produces a fixed-size, rotation- and translation-sensitive feature vector, which characterizes the “view” of the trajectory from the perspective of the reference point. We concatenate the features obtained from each reference point in this way in order to form the final feature vector of the trajectory. The dimension of the feature vector is equal to $N\times (n_{d}+n_{p})$, where *N* is the number of reference points and $n_{d}$ and $n_{p}$ are the number of distance bins and point bins for the MiPo features.
**Algorithm 1** Fixed-MiPo feature extraction algorithm**Require:** Set of *N* reference points $\left\{r_{i}\right\}$, trajectory $T=\{p_{j}\}$**Ensure:** Fixed-MiPo feature vector for *T*, FixedMiPo(T)1:  FixedMiPo(T)←[]2:  **for** each reference point $r_{i}$ **do**3:      Initialize bins for angles relative to the north–south axis4:      **for** each point $p_{j}$ in trajectory *T* **do**5:          Compute distance $d(p_{j},r_{i})$6:          Compute angle $\theta (p_{j},r_{i})$ w.r.t. north–south axis7:          Assign (d,θ) to corresponding angle bin8:      **end for**9:      For each bin, compute average distance and number of points10:    Concatenate bin features into $\mathrm{FixedMiPo}(r_{i})$11:    Append $\mathrm{FixedMiPo}(r_{i})$ to FixedMiPo(T)12:**end for**13:**return** FixedMiPo(T)

**Figure 2:**
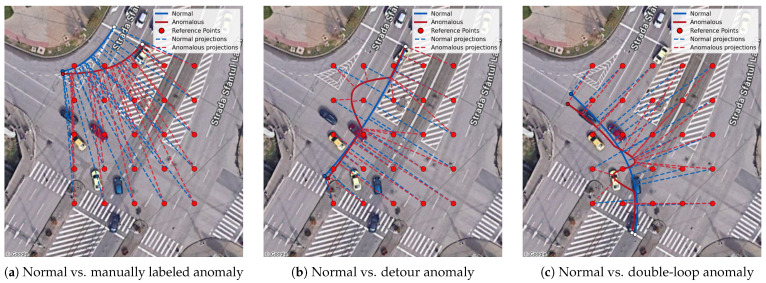
Illustration of LDF unsigned distances for sample normal and anomalous trajectories. Shape deviations change the distances to the nearest reference points, altering the feature vector, which allows them to be detected.

The essentials of the two methods are illustrated in Figure 1. Furthermore, Figure 2 illustrates in more detail how anomalous trajectories alter the LDF feature representation by changing the distances to the nearest reference points.

#### 2.3.3. Landmark Placement

Both proposed solutions rely on a set of fixed reference points, and their number and location are important parameters. We use fixed reference points in order for the resulting features to be sensitive to the absolute spatial placement of trajectories and not just their shape. This is particularly relevant at the intersection level, where vehicles are less constrained in their movement.

We consider several possible landmark placement strategies:

- Uniform grid: The reference points are placed on a uniform N×N grid, spanning the intersection area. The rationale is that reference points need to be close to all areas where trajectories may exist so that any deviation is better reflected in changes of distances, even if an anomalous trajectory is far away from the normal areas. The number of points per row/column is a hyperparameter to be optimized.
- Uniform grid, rotated: The grid can be rotated at various angles to better align with street directions, in the case of rectangular intersections, as an effort to bring the reference points closer to expected trajectory shapes. However, as we shall see in the experiments, the angle does not have a substantial impact.
- Random sampling: The reference points are sampled uniformly at random in the intersection area.
- Uniform + Jitter: Starting from a uniform grid, each point is randomly perturbed by a controllable amount to introduce a certain degree of randomness. We include this strategy as a way of breaking possible unwanted correlations between distances to the landmarks.

We avoid placing the landmarks close to the intersection’s geographical margins because they are less capable of providing discriminative information for characterizing trajectories situated farther away. Instead, we take the bounding box of all the trajectories, and we place the landmarks only within the range [0.2, 0.8] of the width and height in order to keep them relatively close to the central area of the intersection.

### 2.4. New Dataset for Intersection-Level Trajectories

**Figure 3:**
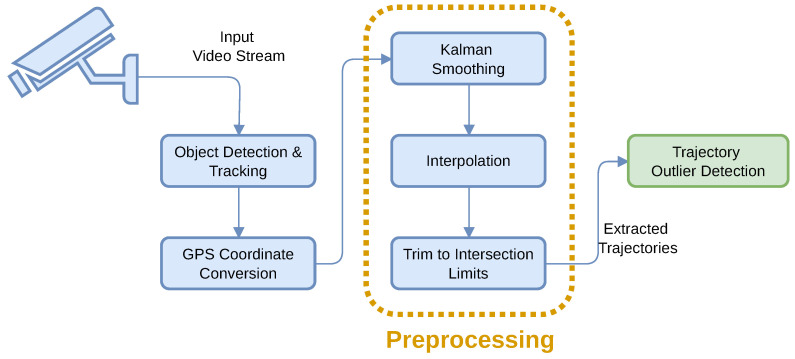
The stages of the trajectory extraction pipeline.

To evaluate our setup, we created a new dataset from real-life recordings in the intersection of Podu Roș, Iași, Romania, where the system is deployed. It consists of 5378 vehicle trajectories, with a total of 1,608,906 trajectory points, obtained from an approximately 165-min video session recorded at 25 frames per second (FPS), using AI-based object detection and tracking followed by a sequence of preprocessing steps. Acquisition setup details are provided in Table 1, and the complete trajectory extraction pipeline is described below and illustrated in Figure 3. We refer to this dataset as the Podu Roș dataset.

**Figure 4:**
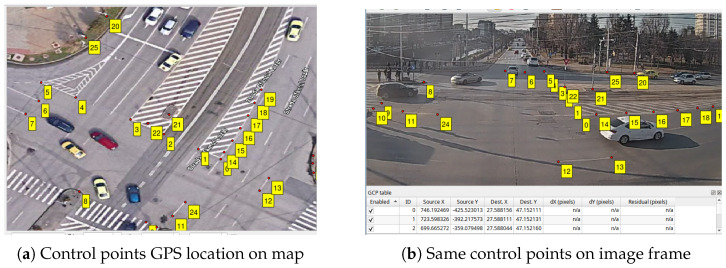
Camera calibration procedure: matching GPS and pixel coordinates for control points (marked with yellow numbers) using QGIS geographic software. (**a**) control points’ positions on the map; (**b**) corresponding positions on the image.

**Figure 5:**
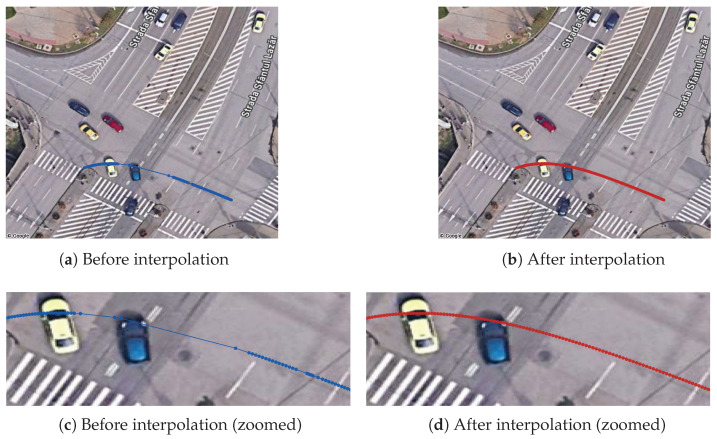
Trajectory interpolation example, overlaid on a Google Maps image. Top row: full trajectory. Bottom row: zoomed on one gap. Original trajectory is shown in blue, interpolated trajectory is shown in red.

1. Object detection and tracking: The first stage in the trajectory pipeline is the object detection and tracking from the source video stream. We use a YOLO v7 model pretrained on the COCO dataset, which is capable of detecting different classes of vehicles, as well as pedestrians. The real-time inference with the model is achieved by using the Savant-AI software framework (version 0.2.11; BWSoft Management LLC, Sheridan, WY, USA; available at https://github.com/insight-platform/Savant, accessed on 19 May 2024), which is a high-level Python framework for computer vision built on top of the NVIDIA DeepStream SDK optimized libraries for inference. As tracker, we use the DCF tracker available in the DeepStream SDK (NvDCF), which provides a balanced speed and accuracy for tracking objects. From a total of 15,676 tracked objects (2,452,102 trajectory points), we selected the 11,573 tracks corresponding to the “car” class (2,114,806 trajectory points) and ignored other classes (vehicles like buses/trams, which follow a predefined route, and pedestrians).
2. GPS coordinate conversion: The trajectories undergo conversion to GPS coordinates before any further processing. First, from each detected 2D bounding box, we take a single point to approximate the vehicle’s ground-plane position, which is then converted to GPS coordinates and added to the trajectory. The point is chosen empirically at 50% width and 10% height of the bounding box to account for the inclined viewing angle.
  Conversion to GPS requires a one-time offline calibration stage in order to determine the intrinsic and extrinsic parameters of the camera, which is achieved using the method introduced in [40], implemented in the *cameratransform* Python package (version 1.2; Richard Gerum; available at https://github.com/rgerum/cameratransform, accessed on 1 June 2025). It relies on georeferencing a set of visible ground control points (matching visible points in the image to their GPS locations), which is manually done using GIS software, as illustrated in Figure 4. The package then uses Monte Carlo sampling to estimate or adjust camera parameters (elevation, heading/tilt/roll angles, and lens distortion model parameters), enabling seamless conversion between pixel and GPS coordinates. The full set of camera parameters obtained is available in Table A1.
  The calibration procedure is fully remote and relatively easy to perform in urban intersections, where there are many control points visible (e.g., roadside poles and street markings), but it is only applicable for fixed views. The resulting accuracy depends on many factors, i.e., number of control points and distance to the region of interest, but deteriorates towards the edges of the field of view due to lens aberrations as well as perspective distortions. Based on visual inspection, we consider the accuracy of the conversion process to be less than 1m in the central region of interest.
3. Kalman smoothing: Trajectory smoothing is a critical preprocessing step since object detection tends to produce noisy trajectories, especially for distant vehicles. We use a Kalman filter with a constant velocity model; the smoothing parameters were optimized via manual inspection in order to achieve a reasonable trade-off between noise reduction and preserving the shape.
4. Interpolation: Trajectories may have gaps due to occlusion by larger vehicles or simply due to object detection failing to recognize objects in certain frames. To obtain consistent trajectories, we interpolate the missing points using GeoPandas. As the data has a fixed time step, given by the video FPS (25 FPS, 40 ms time intervals), we insert the missing timestamps, and we use GeoPandas’ quadratic interpolation to estimate the positions. An example is shown in Figure 5.
  All trajectories in the dataset had at least one missing point to be interpolated, but the vast majority are very small: 89.8% of all such gaps are less than 0.25 s long (i.e., six consecutive points at 25 FPS), 96.2% are less than 0.5 s (twelve points), and no gap is larger than 2.14 s. In total, 213,321 points from a total of 1,608,906 are interpolated (13.3%).
5. Trimming to intersection limits: Finally, the trajectories are trimmed to keep only the part where they actually cross the intersection area, which is our area of interest. Trimming is achieved by manually defining a set of ingress and egress zones, through which the vehicles are expected to enter and exit the intersection areas, and trimming trajectories to these zones.
  As a final step, we keep in the dataset only complete vehicle trajectories, which intersect an ingress and an egress zone. In this way, we filter out possible partial and incomplete trajectories caused by tracking and object detection failures (e.g., trajectories that start or end in the middle of the intersection area because the tracker lost the object), resulting in 5378 full trajectories kept out of 11,573. We have two rationales for this. First, these incomplete trajectories depend primarily on the quality of the AI-based detection and tracking algorithms, which we consider a separate issue, outside the scope of the paper. Second, they would be “easy” anomalies, as they stand out due to their short length and uncommon endpoint location, thus obscuring more subtle anomaly types. By keeping only full-length trajectories, we obtain a curated dataset that is more relevant for feature algorithm comparison. However, a robust solution to handle these incomplete trajectories remains a critical aspect of any practical system deployment.

The trajectories are manually annotated for anomaly detection by a single human expert familiar with the intersection traffic rules, considering both shape and placement abnormalities, using the following protocol.

First, each trajectory was displayed on its own, overlaid on the intersection map, in order to observe its shape and also to understand the ingress and egress roads and lanes taken (each road in this intersection having three lanes). The annotator was instructed to evaluate the spatial deviation of the trajectory from the expected ingress-to-egress movement pattern, considering both irregularities in shape (e.g., abnormal bendings) and uncommon positioning or maneuvers (e.g., taking a left turn starting from the rightmost ingress lane, or doing a U-turn). The scale of the deviation was evaluated and assigned into one of three categories (lane/street/intersection scale), according to the following guidelines, resulting in an integer anomaly level ranging from 0 to 3:

- Level 0: no anomaly.
- Level 1: lane-level trajectory anomalies, i.e., approximately within a lane. Example: an uncommon curvature of the trajectory within a lane.
- Level 2: street-level trajectory anomalies, i.e., anomalies that span multiple lanes within a street. Examples: an abrupt change of lanes or changing from lane 1 to lane 3.
- Level 3: intersection-level trajectory anomalies, i.e., anomalies that span whole streets. Examples: vehicles doing illegal U-turns, crossing over forbidden areas, and driving in the wrong direction.

In a second pass through the dataset, each trajectory with a non-zero anomaly level was reviewed, being displayed on the map together with its closest 10 trajectories, selected with the Hausdorff distance. The goal is to display the trajectory in the context of similar-looking trajectories and give the annotator a chance to reassess the anomaly level in this context. This step is particularly important since trajectories were analyzed sequentially, and similar shapes may appear at much different times.

In case of ambiguous trajectories, as a general rule, the lower anomaly value was preferred.

We acknowledge that this labeling procedure is to some extent subjective, since it was done by a single annotator, and it is based on expected ingress-to-egress movement patterns, not just on explicit traffic-rule violations. However, it also allows capturing a wider range of uncommon trajectory patterns, which may not be traffic-rule violations per se. Furthermore, to reduce the ambiguity, in all our experiments, we simplify the levels to binary: Anomaly levels 2 and 3 are grouped together as the anomalous class, while levels 1 and 0 are grouped as the normal class. This results in a contamination rate of 1.52% (82 anomalies out of 5378), which is towards the lower end of values in literature and thus reasonably conservative.

The annotation procedure can be replicated on other intersections, but some familiarity with the local intersection layout and traffic flow is needed. This is because annotators must not only be aware of the lane geometry and traffic rules but also understand the typical crossing patterns at the lane level in order to assess how a trajectory deviates from the typical ingress-to-egress lane pattern. Therefore, ideally, the annotators should have first-hand familiarity with the intersection.

**Figure 6:**
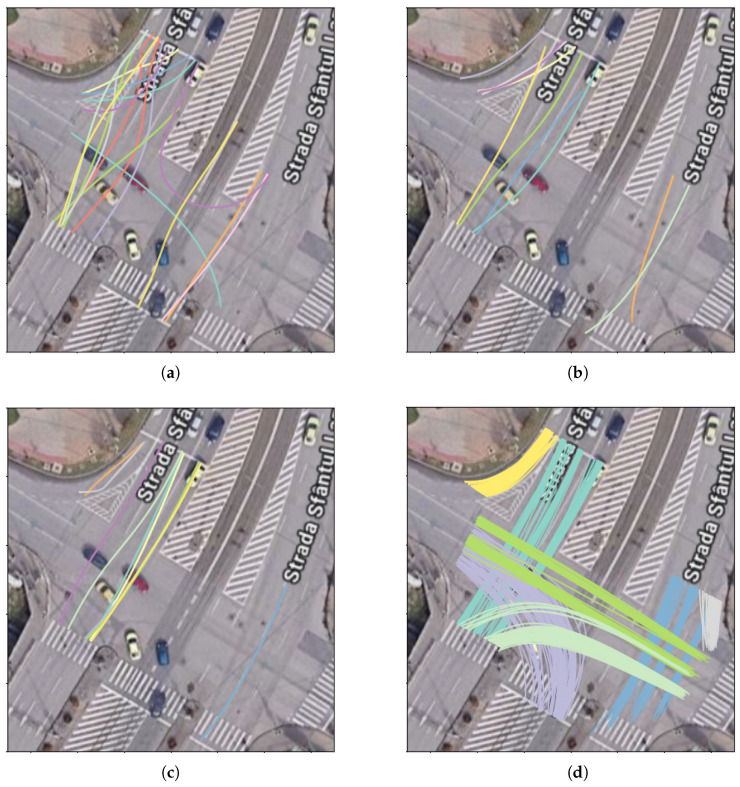
Sample trajectories with different anomaly levels from the dataset. (**a**) Trajectories with anomaly level 3 (intersection-level anomaly). (**b**) Examples of trajectories with anomaly level 2 (street-level anomaly). (**c**) Examples of trajectories with anomaly level 1 (lane-level anomaly). (**d**) Examples of trajectories with anomaly level 0 (no anomaly).

**Figure 7:**
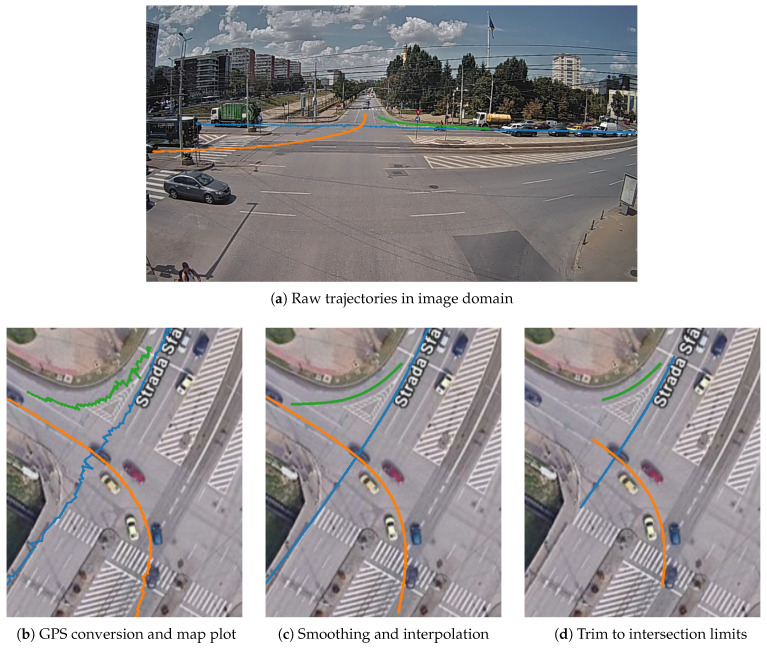
Preprocessing steps for the Podu Roș dataset, illustrated on a few sample trajectories (different colors). The trajectories are detected in the image space, then undergo GPS conversion of coordinates, smoothing, interpolation, and trimming of superfluous margins, keeping only the part that crosses the actual intersection area.

Trajectories from the dataset are illustrated in Figure 6. A summary table with the trajectory classes is presented in Table 2. An illustration of the preprocessing steps is shown in Figure 7.

## 3. Results

We present the results obtained in several experiments, focusing on the following issues:

- How do the proposed methods compare to existing ones for AI-extracted intersection-level trajectories (Podu Roș dataset) as well as for GPS-collected city-wide trajectories?
- Which preprocessing steps and which landmark selection parameters are most important for the proposed methods?
- How do the proposed methods perform with limited training data, as is often the case in online settings?

We use several datasets, as described in Section 3.1.

The algorithms used in the experiments are listed in Table 3. LDF and Fixed-MiPo are the two methods proposed here. The first reference algorithm is from [3], using geometrical features extracted from the trajectories: end-to-end distance between endpoints, total distance traveled, the ratio of these two distances, total curvature, total turning, radius of gyration, coordinates of trajectory centroid, convex hull area, perimeter, centroid, and aspect ratio. The second approach is the MiPo algorithm from [4], and the third is the IKAT method [41], which is based on using a specially crafted Isolation Kernel for trajectory data. Unlike all the others, IKAT does not allow for separate feature extraction and outlier detection stages, since it combines both in a single method.

For all methods except IKAT, we use several common outlier detection models from the PyOD library [42] (version 2.0.5; Yue Zhao; available at https://pyod.readthedocs.io, accessed on 15 June 2025). Following a preliminary experiment, we selected seven models that consistently provided good results: Gaussian Mixture Model (GMM), Angle-Based Outlier Detector (ABOD) [43], K-Nearest-Neighbors (KNN), Local Outlier Factor (LOF), Isolation-based anomaly detection using Nearest-Neighbor Ensembles (INNE), Feature Bagging with LOF base model, and Locally Selective Combination of Parallel Outlier Ensembles (LSCP; using LOF, KNN, and Clustering-Based LOF as base models). We use these seven estimators in all experiments, with all algorithms. In particular, for the GMM model, we set the number of components to seven, which is the number of valid road-to-road crossings of the intersection according to the traffic rules in place.

The models are unsupervised; the trajectory anomaly labels are used only for evaluation, not during training. In particular, KNN is implemented in the PyOD library as an unsupervised outlier detection algorithm, returning the distance to the *k*-th neighbor as the anomaly score.

Our main evaluation metric is the Precision–Recall Area Under the Curve (PR-AUC). For anomaly detection, PR metrics are more relevant than Receiver Operating Characteristic (ROC) (used, for example, in [4]), due to the significant imbalance between outlier and normal classes, which artificially reduces the false positive rate, thus inflating ROC values.

The experiments use a nested stratified K-Fold cross-validation strategy with K=5. This means each dataset is split into five; each part is used in turn as a test set, while the remaining data is again split into five subsets and rotated as train and validation sets in an inner loop. The splits are maintaining the anomaly contamination ratio (stratified). The averaged PR-AUC on the validation sets is used to select the best hyperparameter combination, and the corresponding averaged PR-AUC on the test sets is reported in the tables.

In order to obtain the best results with each method, we run different configurations of the algorithms with multiple parameter values, such as the number of distance bins and point bins for MiPo and Fixed-MiPo, types of distance functions for LDF, number of estimators and samples for IKAT, and number and arrangement of reference points. The complete experiment configurations are too extensive to be listed here but can be inspected in the code repository (see Data Availability at the end of the manuscript). Even though we searched for optimal parameters for each method, amounting to thousands of different configurations, we do not claim to have found the absolute best values. However, the general comparative trends emerging from the data should still be valid.

All the experiments are run offline, on a computing server having two Intel Xeon Silver 4210 CPUs (40 virtual cores), 409 GiB RAM, and an NVIDIA Tesla V100 GPU with 32 GiB memory. The video stream was acquired with a Mobotix Move bullet camera, mounted on a traffic pole in the Podu Roș intersection as part of a broader effort to advance smart city initiatives in partnership with the local municipality.

### 3.1. Datasets

There are very few publicly available datasets with manual annotations of anomalous trajectories [1]; two papers state explicitly that there are no such datasets ([44] in 2020 and [4] in 2022). To alleviate the scarcity of ground-truth information, besides our manually labelled Podu Roș dataset, we extend the evaluation by using synthetically generated perturbations applied to real trajectories, as well as empirical anomaly selection on other datasets, as explained below. These synthetic datasets serve as supplementary evaluation data for the algorithms under controlled conditions.

Podu Roș: We use our Podu Roș dataset in two ways: with the manually annotated anomalies as described in Section 2.4 or with synthetically generated perturbations applied to real trajectories, following the approach in [1,4,19,20,44]. Inspired by the implementations in other works, we generate five different types of localized spatial perturbations, listed in Table 4, which are applied to randomly selected trajectories. For each perturbation, we consider three degrees of severity (small/medium/large), and we save the dataset separately, resulting in a total of fifteen perturbed datasets based on the original Podu Roș data (5 perturbations × 3 severities).

The five perturbation models aim to simulate various types of abnormalities. Detour, route switch, and point shifts have been used in literature, e.g., [4,19,44]. Detours and double bends are one-sided and two-sided bendings aiming to simulate sinuous driving; route switches simulate new movement patterns by splicing segments from existing trajectories, and shortcuts mimic cutting corners of rounded maneuvers. Point shifts are basic one-point aberrations.

**Figure A1:**
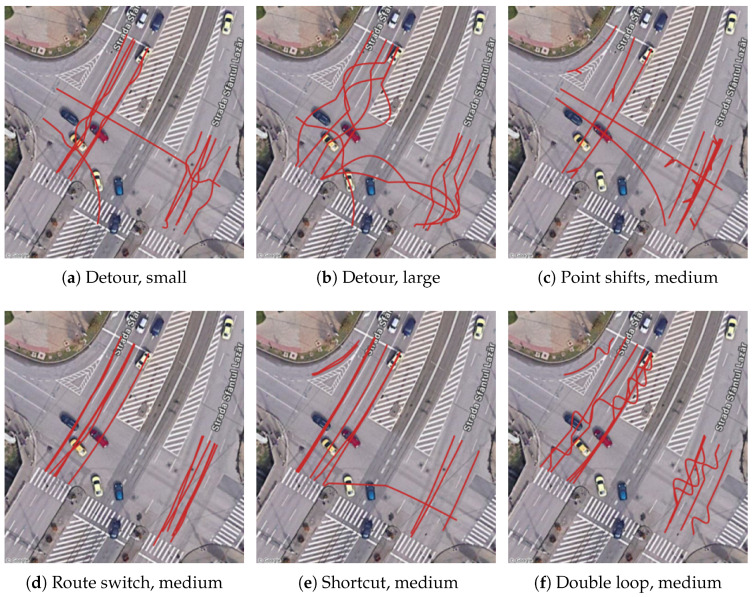
Generated perturbation types for the Podu Roș dataset, illustrated on a few sample trajectories.

**Figure A2:**
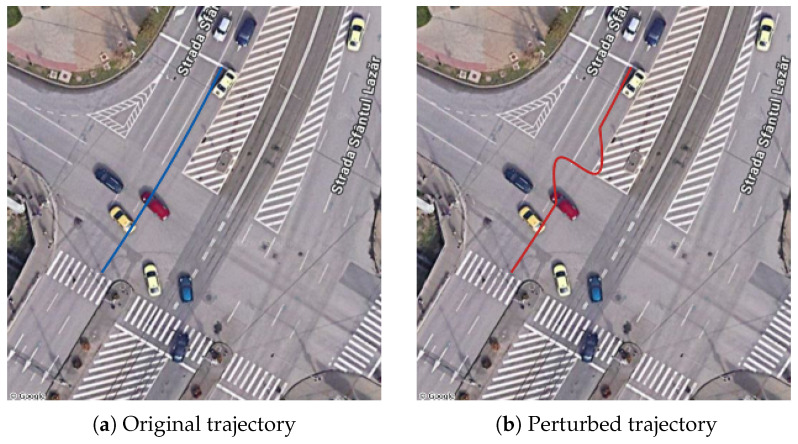
Trajectory before (blue) and after (red) perturbation (double loop, large).

We set a contamination ratio of 5%; the perturbed trajectories are randomly selected for every combination. From each selected trajectory, we randomly select a segment of configurable length, which undergoes a spatial modification: For detours and double bends, we add to it a spatial sinusoidal component (one-sided or two-sided); for shortcuts, we replace it with a straight segment; and for route switches, we replace it with a random segment taken from another trajectory, properly scaled and rotated. For point shifts, a random trajectory point is displaced by a controlled amount in a random direction. The perturbations are illustrated in Figure A1, while Figure A2 shows a single trajectory before and after perturbation.

Porto: We also use the labeled city-wide trajectory datasets from [4], which are based on the Porto Taxi 2015 dataset [28] (available here: https://github.com/TimeIsAFriend/trajectory_MiPo, accessed on 1 September 2025). These consist of 9044 trajectories extracted from the larger Porto dataset, with different types of synthetically generated shape anomalies (detour, point shifts, and route switching) and organized into 12 smaller datasets. The trajectories with injected anomalies are considered anomalous, and the remaining ones constitute the normal class. The complete datasets, including the perturbed trajectories and the labels, are inherited from [4]; we simply reuse them as is. Note that these trajectories are not collected from intersections nor extracted with AI tools from video data but rather are general city-wide GPS-collected trajectories. We do not apply any preprocessing to these trajectories; we use them as they are. Therefore, they are representative of a much larger class of trajectories than the Podu Roș dataset, which is specialized in short intersection-level trajectories.

Hannover and Edessa: Another two datasets with city-wide GPS-collected trajectories in the cities of Hannover and Edessa are available from Leibniz University Hannover [45,46]. While they are not annotated for anomaly detection, the authors include a detailed shapefile with the road junctions, with GPS location and ingress/egress junction arms, which allows for an empirical estimation of anomalous behavior in intersections. We take the following approach. For each trajectory, we find the junctions through which it passes based on proximity to the junction coordinates; we estimate the entry and exit angles in these junctions; and we match them to the closest arm angles available in the junction description. We define an anomalous event when the difference between the trajectory entry/exit angle in a junction and the matched arm exceeds a threshold, i.e., when the trajectory appears to come from an unknown direction, or when a particular entry/exit route through the junction is taken very rarely among all the trajectories that cross that junction (less than 0.5% of the total). Every trajectory that contains such an anomalous event is considered an anomalous trajectory. All the other trajectories are considered the normal class. In the end, this labeling procedure selects, for Hannover, 82 anomalous trajectories out of a total of 1181, and, for Edessa, 103 anomalous trajectories out of 341.

In total, there are thirty different dataset configurations on which we run the evaluations: the Podu Roș dataset with manual labels, fifteen Podu Roș variants with synthetic perturbations of different severity, twelve Porto datasets with synthetic perturbations, and two datasets (Hannover and Edessa) with empirically selected anomalies.

We point out that the datasets cover different application scenarios. Only the manually labeled Podu Roș data directly validates the proposed methods in the context of video-based intersection monitoring. The synthetic Podu Roș variants provide supplementary evaluation for the same intersection-level trajectory domain, using controlled amounts of perturbations. The Porto, Hannover, and Edessa datasets are complementary city-wide datasets, constituting a different application setting, but we included them in our analysis for multiple reasons. Our primary intersection use case is narrow, focusing on a single site, with other intersection-level datasets scarcely available; this limits broader validation and acceptance. In addition, as most research in this field revolves around larger-scale trajectories, we are interested in evaluating whether the proposed features are particularly adapted to an intersection setting or can also be used with more mainstream datasets. Lastly, the Porto datasets were used in prior work on the MiPo features [4], while the Hannover and Edessa datasets come with additional junction geometry metadata, allowing us to empirically identify anomalies with respect to the intersection layout; thus, these datasets constitute suitable benchmarks. However, we point out that the Porto, Hannover, and Edessa datasets provide complementary evidence on the flexibility of the feature representations in a broader context without directly supporting the primary target use case of video-based intersection monitoring.

We use the same procedure for computing the features for city-wide trajectories as for intersection-level data. For the proposed features, the reference points have no particular relation to the underlying map information; we determine the bounding box encompassing all the trajectory data, and within the bounding box, we pick the reference points according to the configured placement strategy (e.g., uniform grid, jittered, or random selection). The distance-based features are computed with respect to these reference points, as described in Section 2.3.

### 3.2. Statistical Feature Analysis

To better understand the feature distributions across anomaly classes, we conduct a brief statistical analysis of the features for the Podu Roș dataset with manual labels. Specifically, we consider t-SNE embedding visualization and LDA-based analysis for the LDF features obtained using 49 reference points (i.e., feature vectors of length 49) arranged in a 7×7 grid, with no rotation, and using unsigned distances.

**Figure 8:**
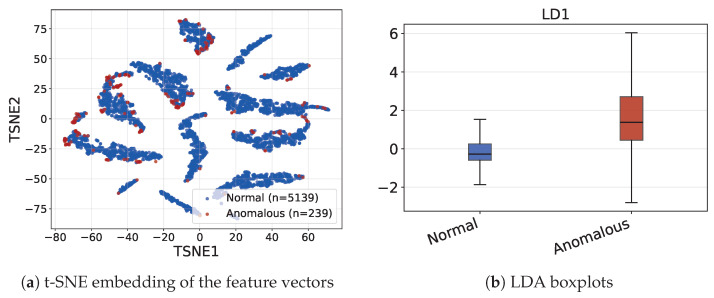
Statistical feature analysis for the LDF features on the Podu Roș dataset (49 reference points).

In Figure 8a, we show the 2D t-SNE embedding of the feature vectors. The highly clustered nature of the embedding likely reflects the natural spatial clustering of trajectories in clear street-to-street crossing patterns, as visible also in Figure 6d. The anomalous trajectories appear as fringe points of several clusters, suggesting they are deformed variants of trajectory patterns.

We also run an LDA separability analysis, considering the binarized anomaly labels used in the experiments (anomaly levels 2 and 3 grouped as the anomalous class and levels 0 and 1 as the normal class). In Figure 8b, we show the boxplots for the LD1 projections onto the discriminant axis, per class label. There is a clear shift in the distributions between the normal class (−0.0879 ± 0.8679) and the anomaly class (1.8894 ± 2.5157), with the ROC-AUC score of 0.8128, which suggests that the features are capturing relevant discriminative information.

We additionally performed a MANOVA analysis on the features to investigate if the feature ensemble differs significantly between the normal/anomaly classes. For stability reasons, we selected only the nine features with the highest variance out of the total of forty-nine. The anomaly class label is the independent variable; as the original four anomaly levels are highly imbalanced, especially for anomaly level 3 (only 17 trajectories), we preferred to use the binarized labels as well. The analysis shows significant differences: Pillai’s is trace 0.0371, F(9,5368)=22.97, and p<0.001.

We ran the same feature analysis for the Fixed-MiPo features (108 features, 9 reference points × 12 MiPo-based features for each); LDA analysis shows similar class shifts along the discriminative axis (normal class LD1: −0.0974 ± 0.8343, anomalous class LD1: 2.0937 ± 2.7500), with an ROC-AUC score of 0.8439. For reasons of brevity, we do not include the images in the manuscript.

### 3.3. Anomaly Detection Results

We present in Table 5 the PR-AUC values obtained on all 30 dataset configurations using a nested stratified K-Fold cross-validation approach. Each row reports, for a given dataset, the average test set results of the best-performing anomaly detection model on the validation sets. The best-performing models themselves, for each dataset and feature type, are listed in the appendix in Table A3. For the Porto datasets, we keep the original naming from [4] (AiSq5D to UnCh10M). Note that the values of MiPo for the Porto datasets are different from those reported in [4], since they report only ROC-AUC, while we report PR-AUC, which we consider more appropriate for anomaly detection tasks.

For the manually annotated labels of the Podu Roș dataset, we consider the anomaly levels 2 and 3 as the outlier class, with levels 0 and 1 being the normal class.

The results show that the proposed LDF method achieves substantially larger PR-AUC values than the other methods in virtually all scenarios considered. Fixed-MiPo, while achieving the second-best score on average, has a more nuanced standing: While it is a clear second-best on the manually labeled Podu Roș dataset, it is closer to the results of Geometric and MiPo for other datasets. Note that IKAT yields substantially lower PR-AUC values than all the others. We could not find a parameter set for IKAT with better results.

The comparative ranking of the features remains largely similar for intersection-level data (Podu Roș) and for the city-wide datasets (Porto, Hannover, and Edessa), despite the different nature and spatial scale of the trajectory data, with LDF providing the best scores on most datasets and Fixed-MiPo generally being in second place or close to it. In contrast, a clear shift is visible in the outlier detection models corresponding to these scores, listed in Table A3: For intersection-level datasets, KNN and GMM are among the top-performing models, whereas ABOD emerges as the more preferred model for city-wide datasets, likely reflecting the larger variability in city-wide data compared with the clustered data of a single intersection site. This suggests that the choice of the detection model, rather than the feature representation, is more sensitive to the nature of the trajectory data, with the proposed features still capturing discriminative information even from large-scale data.

We applied the Wilcoxon two-sided signed-rank tests, using Holm–Bonferroni correction, on pairs of algorithms, considering the 30 test results reported in Table 5 for all the datasets. Considering a significance level of 0.05, all algorithm comparisons are statistically significant, except for the pair of reference methods, MiPo and Geometric. The numerical analysis results are provided in Table A2.

### 3.4. Best Tabular Outlier Detection Models

A natural question is which tabular detection model performs best in the experiments. Even if the final choice might well depend on the dataset, feature type, or other factors, we would still like to have a shortlist of good candidates to consider for future applications.

To achieve this, we derive an empirical model ranking, based on the top results of every model, as follows. Considering that we used thirty dataset variations in the evaluation and we used eight anomaly detection models (seven from PyOD plus IKAT), there are 240 (dataset, model) pairs. For each (dataset, model) pair, we select the best-performing result across all feature types (LDF, Fixed-MiPo, Geometric, MiPo, and IKAT), according to the validation scores. Then we compute the average test AUC-PR per detection model, and we count how many times each model is present in the top-1 and top-3 results of each dataset. The ranking is presented in Table 6, where “Top1-Count” indicates how many times a model is the best-performing one for the 30 datasets, “Top3-Count” indicates how many times a model is present in the top-3 results of the 30 datasets (3 models × 30 datasets = 90 positions), and “Mean Test PR-AUC” indicates the corresponding average test scores.

Table 6 shows GMM and ABOD to be the best-performing models across these datasets, followed by KNN. The mean PR-AUC of GMM and ABOD are very close, showing that the choice among them is not critical.

The top models for each individual dataset and feature type are listed in Table A3. Interestingly, GMM outlier detection shows good results on Podu Roș datasets but poorer results on Porto, Hannover, and Edessa. One possible reason is that the intersection trajectories are naturally clustered in street-to-street crossing patterns, and when GMM is initialized with an appropriate number of components, it succeeds in capturing this structure. For the city-wide trajectories, the structure is more complex and does not lend itself to good clustering with GMM.

**Figure 9:**
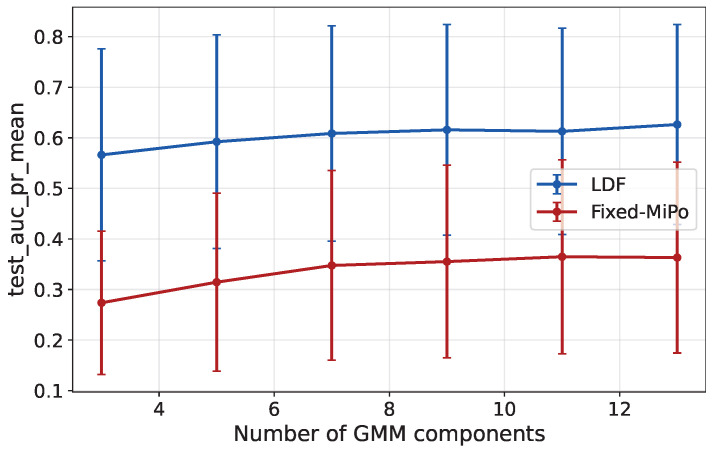
Average test results with GMM for Podu Roș datasets for different numbers of components. GMM is not particularly sensitive to this parameter, as long as the value is reasonably large.

We investigate whether the GMM results are influenced by the particular value of the number of components in a separate experiment. Figure 9 plots the average results obtained with GMM on all Podu Roș datasets (manual and generated anomalies) as a function of the number of components, with the other hyperparameters fixed to the best values among all experiments on these datasets. The figure shows that the number of components is not a critical parameter, as long as it is a reasonably high value.

### 3.5. Landmark Selection

Both LDF and Fixed-MiPo algorithms rely on a set of reference points (landmarks) scattered through the intersection, and their number and arrangement are hyperparameters that we would like to investigate. To find out which values are important, we use the following approach. For each algorithm, we select the top-performing parameter combinations on the Podu Roș dataset, with a PR-AUC value within 0.05 of the best, and we establish the frequency of each parameter value to identify which values are consistently associated with top results. We perform this analysis only on the Podu Roș dataset with manually annotated anomalies.

For the LDF algorithm, we have 94 parameter combinations within 0.05 of the best PR-AUC. We do not report the exact data for each combination for brevity reasons, but we summarize below our conclusions.

- Among the ninety-four parameter combinations, the number of landmark points used is almost uniformly spread between nine points (22/94 combinations) and one hundred points (14/94 combinations). Thus, the number of points does not seem to be a critical parameter, as long as it is in a reasonable range.
- The arrangement of landmark points strongly favors a square grid (40/94 combinations), possibly with some limited degree of randomness added (38/94), but not purely random (6/94).
- As to rotation, we have not observed any important relation to the intersection topology. Most of the combinations are not rotated (37/94), which makes them not aligned with the main roads. As a consequence, we cannot say that the rotation plays a clear role.
- Distance measures: all the combinations use unsigned distances (i.e., Euclidean) and not signed distances.

For Fixed-MiPo, there are only five parameter combinations within 0.05 of the best PR-AUC. The majority use nine reference points (4/5 combinations), randomly selected in the intersection area (4/5 combinations), as opposed to a regular grid or corner location.

### 3.6. Ablation Study for Preprocessing Steps

The trajectories in the Podu Roș datasets featured in the experiments undergo three major preprocessing steps: Kalman smoothing, interpolation, and trimming. We investigate the relevance of each of these steps via an ablation study, in which we enable them consecutively and evaluate the results, as reported in Table 7. We perform the analysis only on the Podu Roș dataset with manually annotated anomalies.

The results in Table 7 show that LDF features provide the best results for all types of preprocessing except raw trajectories, where they are surpassed by MiPo. We also note that the best outlier detection model generally changes depending on the feature type and on the level of preprocessing, with ABOD being the most often encountered.

We note that preprocessing brings a major benefit in results, especially for LDF and Fixed-MiPo. As a consequence, their results on the Podu Roș trajectories should be considered as resulting from both preprocessing design and feature design. In particular, the table shows that trimming is the preprocessing step with the greatest impact, most likely due to the increased trajectory noise and variability towards the endpoints, caused by the limitations of the camera view setup and the increased object detection errors in distant areas. Trajectory smoothing has a moderate benefit for all but the MiPo-based algorithms, while the effect of interpolation is small.

### 3.7. Sequential Outlier Detection

As a final experiment, we consider a sequential outlier detection approach, in which the anomaly detection models are trained on a sliding window of recent trajectories and are used to predict anomalies on the next trajectories. This is motivated by the end goal of using these algorithms in an automatic anomaly detection system operating continuously on live traffic systems when only a limited amount of past data is available.

Specifically, we consider the Podu Roș dataset with the manually annotated labels, split into overlapping frames of 512 trajectories, with a stride of 128. The models are trained on a single window and are used to predict anomalies on the next 128 trajectories; then the window shifts, the models are retrained, and the process is repeated. After processing the whole dataset, we gather all the anomaly scores, and we compare them against the ground-truth labels. This is a simplified setup that avoids the practical issue of selecting separate thresholds per window but allows us to obtain a global view of the results against ground truth for a proper evaluation.

This sequential procedure is implemented using the PySAD framework (version 0.3.4; available at https://pysad.readthedocs.io, accessed on 15 September 2025), wrapping around the anomaly detection models implemented in the PyOD library. In this experiment, we skipped the IKAT algorithm due to implementation efficiency.

The results are reported in Table 8. Again, LDF features provide the best results, followed by the Fixed-MiPo approach. There is a drop in PR-AUC values for all feature types used, highlighting the impact of reduced training data; e.g., the PR-AUC for LDF drops to 0.472 (from 0.590 on the full dataset). ABOD remains the most appropriate detection model for most feature types.

## 4. Discussion

### 4.1. Interpretation of the Results

We discuss the relevance of the results from three perspectives: (i) what types of features are more appropriate for spatial intersection trajectory anomalies; (ii) what outlier detector models provide the best results; and (iii) whether the methods are capable of continuous online operation.

First, all the results presented throughout Section 3 indicate that the LDF distance-based features are better suited than the alternatives for anomaly detection of trajectories for both intersection-level and city-wide trajectories. Our second proposed algorithm, Fixed-MiPo, is second best for the Podu Roș dataset with manually annotated labels, but on the other datasets, it is generally competing with MiPo or Geometric for second place.

The better results of landmark-based methods for intersection-level trajectories may be explained as a combination of both the preprocessing and feature design: The preprocessing we apply to the Podu Roș data (i.e., smoothing, interpolation, and trimming) produces trajectories with a smoother shape, located in a well-defined region of interest, making them well described by distances to landmarks. The ablation study shown in Section 3.6 confirms that the preprocessing steps have an impact on all algorithms, but especially on the proposed landmark-based features, with trimming being the most important step. As such, preprocessing is a critical component in our intersection results.

In terms of robustness, we highlight that LDF achieves top results with a large variety of datasets, with both intersection and city-wide trajectories, and with either generated, manually annotated, or empirically selected anomalies, making it a good feature choice when only spatial trajectory information is used. Moreover, our analysis in Section 3.5 indicates that LDF is not particularly sensitive to the number of reference points as long as they are in a reasonable number and well spread across the map. In terms of robustness to trajectory noise, the ablation experiment shows that both LDF and Fixed-MiPo benefit substantially from preprocessing. With the raw noisy trajectories, their performance is below MiPo. However, LDF also performs well on the city-wide trajectories in the Porto, Hannover, and Edessa datasets, which undergo no preprocessing. We conclude that LDF and Fixed-MiPo are sensitive to high amounts of spatial noise, as in the case of raw trajectories extracted from video with object detection tools.

Second, in terms of which outlier detection models provide the best results, the picture is more nuanced. While [4] recommended the use of LOF for MiPo features, our model ranking showed that GMM and ABOD generally perform best across various types. Moreover, Table A3 suggests that GMM is a better alternative for the Podu Roș datasets, while ABOD is better for city-wide trajectories. A possible reason might be the clustered nature of intersection trajectories, especially following the preprocessing stages employed, which is well captured by GMM, especially when initialized with an appropriate number of components (i.e., the number of street-to-street crossing patterns). This parameterization of the GMM model is a feasible assumption in a practical deployment of the system. Overall, this is an advantage of intersection-level trajectory anomaly detection, as opposed to more general city-wide trajectories, which follow more complicated patterns.

However, in many cases, the differences between models are small, as visible from the mean PR-AUC scores in Table 6. Furthermore, the preprocessing steps also influence the best choice of model. As a result, observing no definite winner among the outlier detection models, we recommend evaluating several of them in application-specific conditions, with the likely best candidates being ABOD, GMM (if a good initialization is available), and KNN.

Finally, when it comes to practical use in a streaming setting, appropriate for continuous long-term monitoring of live streams, the results in Section 3.7 show a drop in the PR-AUC, with LDF features still providing the best results. Some decrease was expected due to training on a limited window of data, with outlier detection models such as KNN and GMM being sensitive to the amount of training data.

### 4.2. Limitations and Future Work

In the broader context of anomaly detection, we highlight that our work only targets spatial anomalies and therefore cannot capture anomalies in terms of speed, acceleration, or more complex traffic behavior.

A particular limitation of the proposed methods is that they require covering the area of interest with well-spread reference points, which may be impractical for large spatial areas. The density of points determines the scale of the deviations that can be sensed. Sensing small-scale deviations might require an impractical number of reference points, especially when trajectories span larger geographical areas. Moreover, for landmark placement, the area of interest needs to be defined in advance. Thus, landmark-based approaches are particularly suited for video monitoring, which covers a fixed and limited viewing area with dense trajectories. However, in other scenarios, methods that exploit the intrinsic characteristics of trajectories might be more appropriate.

Another critical limitation of our current approach is that it operates with complete trajectories, and therefore, anomaly detection can only be performed after the vehicle has completely passed through the intersection and not during the actual crossing. Investigating the use of sub-trajectories in order to provide an earlier assessment remains an extension for future work.

With regard to evaluation, our intersection data is collected from a single site, and therefore, confirmation on other intersection sites is needed before drawing more general conclusions. In addition, whether the performance achieved in a streaming setting is sufficient for a practical deployment remains an open question.

## 5. Conclusions

In this paper, we investigate spatial trajectory feature representations for intersection-level trajectories extracted from video streams using AI tools. We introduced two feature representations based on distances to reference points, LDF and Fixed-MiPo, and evaluated them using a dataset of 5378 trajectories, extracted with AI tools from traffic video recorded in a busy intersection and hand-labeled for anomaly detection. The proposed features are limited to spatial trajectory anomaly detection and cannot be used to detect temporal anomalies like abrupt speed changes or stopping.

Evaluation is done using data collected from one target intersection site. Our experiments in this setting show that LDF and Fixed-MiPo consistently provide better results than other spatial feature representations on short, dense, intersection-level trajectories extracted from the video data when used with appropriate preprocessing and several common outlier detection models and parameter combinations. LDF also yields better results on general trajectory datasets. We also find that preprocessing plays a major role when it comes to trajectories extracted using AI object detection tools, with trajectory trimming to a well-defined region of interest being the most important step, especially for the proposed methods. However, validation on other target sites is needed before reaching broader conclusions.

The performance of these methods in a streaming setting remains an open question, as we find a drop in PR-AUC when the models are trained on limited amounts of training data across all models tested. Accordingly, future work should focus on validation on additional intersection datasets and on investigating more realistic online training setups.

## Figures and Tables

**Table 1:** Data collections details.

Description	Value
Intersection coordinates	47.15210° N, 27.58801° E
Recording date & time	16 July 2024, 19:09–21:55 (166 min)
Camera model	Mobotix Move bullet camera
Mounting details	Pole above ingress road, elevation ≈ 5.6 m, pitch angle ≈ 75°
Resolution	1280 × 720 @ 25 FPS
AI tools	YOLOv7x detector, NvDCF tracker, NVIDIA DeepStream SDK, Savant-AI framework
AI hardware	NVIDIA Tesla V100 GPU, 32 GiB memory

**Table 2:** Podu Roș dataset summary.

Anomaly Level	Description	Count
0	No anomaly	5139
1	Lane-level anomaly	157
2	Street-level anomaly	65
3	Intersection-level anomaly	17
Total	-	5378

**Table 3:** Algorithms used in the anomaly detection experiments.

Method	Features	Anomaly Detection
LDF	Proposed landmark distance features	Tabular outlier detection models from PyOD library
Fixed-MiPo	Proposed Fixed-MiPo features	Tabular outlier detection models from PyOD library
Geometric features	The set of geometrical features from [3]	Tabular outlier detection models from PyOD library
MiPo	MiPo features from [4]	Tabular outlier detection models from PyOD library
IKAT	IKAT outlier detection method from [41]	IKAT outlier detection method from [41]

**Table 4:** Generated perturbation types on the Podu Roș dataset.

Dataset Name	Anomaly Type	Perturbation Details
Podu Roș detour	Generated	Create a localized lateral bend in a trajectory
Podu Roș route switch	Generated	Replace part of a trajectory with a segment from another trajectory
Podu Roș point shift	Generated	Randomly shift one point of a trajectory
Podu Roș shortcut	Generated	Straighten a part of a trajectory
Podu Roș double loop	Generated	Create a sinusoidal-based double bend in a trajectory

**Table 5:** PR-AUC of outlier detection algorithms on all dataset configurations. Bold values indicate the best result for each dataset configuration.

Dataset	LDF	Fixed-MiPo	Geometric	MiPo	IKAT
Podu Roș manual labels	**0.590**	0.487	0.238	0.213	0.016
Podu Roș detour small	**0.799**	0.369	0.426	0.193	0.063
Podu Roș detour medium	**0.867**	0.483	0.465	0.345	0.061
Podu Roș detour large	**0.892**	0.625	0.518	0.501	0.067
Podu Roș route switch small	**0.385**	0.203	0.134	0.135	0.060
Podu Roș route switch medium	**0.525**	0.203	0.157	0.155	0.067
Podu Roș route switch large	**0.584**	0.255	0.214	0.198	0.074
Podu Roș point shift small	**0.675**	0.231	0.454	0.155	0.064
Podu Roș point shift medium	**0.749**	0.303	0.580	0.174	0.063
Podu Roș point shift large	**0.745**	0.398	0.637	0.186	0.067
Podu Roș shortcut small	**0.353**	0.206	0.135	0.133	0.070
Podu Roș shortcut medium	**0.453**	0.213	0.152	0.145	0.065
Podu Roș shortcut large	**0.476**	0.236	0.183	0.172	0.065
Podu Roș double loop small	**0.872**	0.475	0.566	0.252	0.061
Podu Roș double loop medium	**0.884**	0.585	0.590	0.384	0.063
Podu Roș double loop large	**0.907**	0.666	0.574	0.516	0.064
Porto AiSq5D	0.957	0.984	0.892	**0.990**	0.049
Porto AiSq5DP	**0.945**	0.940	0.807	0.906	0.050
Porto AiSq5P	**0.999**	0.879	0.661	0.881	0.050
Porto AiSq10D	**0.971**	0.957	0.880	0.938	0.100
Porto AiSq10DP	**0.985**	0.942	0.778	0.929	0.100
Porto AiSq10P	**0.998**	0.934	0.623	0.927	0.098
Porto StSt5M	**1.000**	0.977	0.693	**1.000**	0.052
Porto StSt5R	**0.942**	0.760	0.463	0.901	0.046
Porto StSt10M	**0.980**	0.931	0.600	0.945	0.100
Porto StSt10R	**0.953**	0.817	0.597	0.759	0.090
Porto UnCh5M	**0.958**	0.922	0.814	0.872	0.048
Porto UnCh10M	**0.973**	0.927	0.806	0.894	0.100
Hannover Trips	**0.195**	0.158	0.121	0.160	0.069
Edessa Trips	0.582	**0.583**	0.431	0.505	0.302
Average Podu Roș	**0.672**	0.371	0.376	0.241	0.062
Average Porto	**0.972**	0.914	0.718	0.912	0.074
Average Hannover+Edessa	**0.389**	0.370	0.276	0.333	0.185
Average All	**0.773**	0.588	0.506	0.516	0.075

**Table 6:** Empirical ranking of anomaly detection models for all datasets.

Model	Mean Test AUC-PR	Top1 Count	Top3 Count
GMM	0.697065	15/30	16/90
ABOD	0.696437	8/30	26/90
KNN	0.665834	1/30	22/90
LSCP	0.641028	1/30	9/90
FeatureBagging	0.637999	2/30	6/90
LOF	0.637475	1/30	7/90
INNE	0.511560	2/30	4/90
IKAT	0.069268	0/30	0/90

**Table 7:** Ablation study of preprocessing steps on the Podu Roș trajectory dataset: best PR-AUC and outlier detection model for each algorithm. Bold values indicate the best result in each case.

Feature Type	Trajectory Preprocessing Steps			
	None (Raw)	Smooth	Smooth & Interpolate	Smooth & Interpolate & Trim
LDF	0.144	**0.216**	**0.220**	**0.590**
Fixed-MiPo	0.120	0.085	0.077	0.487
Geometric	0.056	0.069	0.082	0.238
MiPo	**0.156**	0.151	0.164	0.213
IKAT	0.018	0.017	0.022	0.016
	**Best outlier detection model for each algorithm**			
LDF	ABOD	ABOD	ABOD	ABOD
Fixed-MiPo	GMM	ABOD	ABOD	KNN
Geometric	ABOD	FeatureBagging	FeatureBagging	GMM
MiPo	FeatureBagging	FeatureBagging	FeatureBagging	ABOD
IKAT	IKAT	IKAT	IKAT	IKAT

**Table 8:** Sequential anomaly detection on the Podu Roș trajectory dataset, using a moving window of 512 trajectories. Bold values indicate the best result in each case.

Dataset	Feature Type	Model	PR-AUC	ROC-AUC	Best F1	Precision	Recall
Podu Roș	LDF	ABOD	**0.472**	0.954	0.456	0.474	0.439
Podu Roș	Fixed-MiPo	ABOD	0.306	0.952	0.378	0.325	0.451
Podu Roș	Geometric	GMM	0.189	0.890	0.306	0.268	0.355
Podu Roș	MiPo	ABOD	0.163	0.919	0.237	0.257	0.220

**Figure A3:**
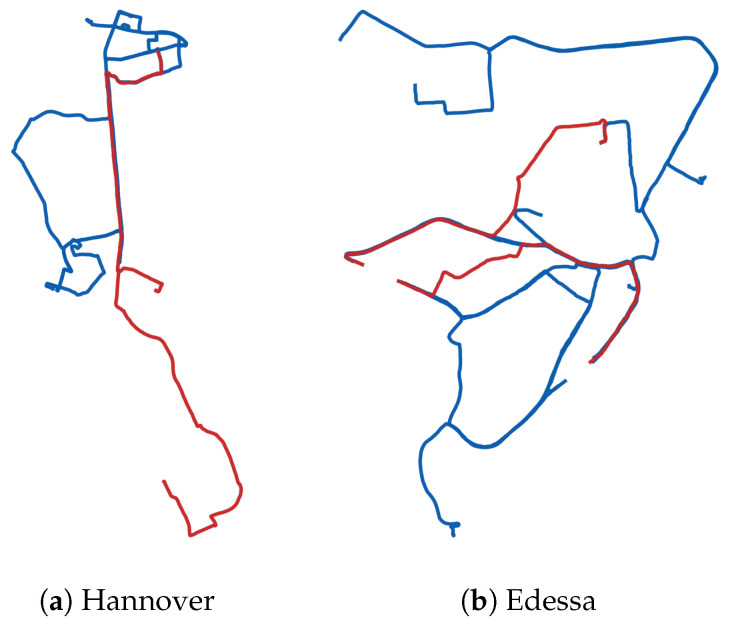
Sample trajectories from Hannover and Edessa datasets: ten normal (blue) and two anomalous (red).

## Data Availability

The code of the paper is available at the following URL: https://gitlab.com/trafalert-project/TrafAlert/-/tree/paperoutlierdet?ref_type=heads (accessed on 30 April 2026). The dataset description and download links are available at the following URL: https://trafalert-project.gitlab.io/TrafAlert/papers/trajoutlierdet.html (accessed on 30 April 2026).

## Appendix A

The appendix contains large figures and tables that would disturb the normal flow of the article.

The complete list of camera parameters obtained after the calibration procedure with the *cameratransform* package is available in Table A1.

**Table A1:** Full set of Podu Roș camera parameters obtained after the calibration procedure.

Name	Value	Description
gps_lat	47.15197798	Camera GPS position (latitude)
gps_lon	27.58837884	Camera GPS position (longitude)
focallength_x_px	709.7420546521115	Focal length X-axis (px)
focallength_y_px	712.8433403950191	Focal length Y-axis (px)
center_x_px	635.4689673768391	Optical image X-axis center (px)
center_y_px	359.35489396854643	Optical image Y-axis center (px)
image_height_px	720	Image height (px)
image_width_px	1280	Image width (px)
sensor_height_mm	4.29	Sensor height (mm)
sensor_width_mm	5.76	Sensor width (mm)
elevation_m	5.636764389174498	Camera mounting height (m)
tilt_deg	79.4546817234508	Camera tilt angle (degrees)
roll_deg	1.99302800280354	Camera roll angle (degrees)
heading_deg	306.3921451394057	Camera heading angle (degrees)
pos_x_m	−2.1432194117299126	Position adjustment (meters)
pos_y_m	−0.19008576507707303	Position adjustment (meters)
k1	−0.17532888886257564	Lens distortion parameter
k2	−0.08617325007201872	Lens distortion parameter
k3	0.04771775705518565	Lens distortion parameter
projection	0	Internal parameter
lens	2	Internal parameter

In Figure A1, we illustrate the five types of perturbations generated on the Podu Roș dataset. Figure A2 shows a single trajectory before and after perturbation.

In Figure A3, we illustrate several trajectories, normal and anomalous, from the Hannover and Edessa datasets.

In Table A2, we list the results of Wilcoxon signed-rank tests for every pair of algorithms. With a significance level of 0.05, all algorithm comparisons are statistically significant, except the pair of the reference methods MiPo and Geometric.

**Table A2:** Wilcoxon signed-rank tests for pairs of algorithms, with Holm–Bonferroni correction (α=0.05).

		*W*	$p_{\mathrm{raw}}$	$p_{\mathrm{Holm}}$	*r*
LDF	Geometric	0	0.0000	0.0000 *	−0.873
LDF	MiPo	2	0.0000	0.0000 *	−0.866
LDF	Fixed−MiPo	6	0.0000	0.0000 *	−0.851
LDF	IKAT	0	0.0000	0.0000 *	−0.873
Fixed−MiPo	MiPo	44	0.0001	0.0003 *	−0.708
Fixed−MiPo	Geometric	93	0.0041	0.0082 *	−0.524
Fixed−MiPo	IKAT	0	0.0000	0.0000 *	−0.873
MiPo	Geometric	213	0.6884	0.6884	−0.073
MiPo	IKAT	0	0.0000	0.0000 *	−0.873
Geometric	IKAT	0	0.0000	0.0000 *	−0.873

Note: * indicates statistical significance after Holm–Bonferroni correction at α=0.05.

In Table A3, we present the best-performing outlier detection algorithms corresponding to the evaluation results presented in Table 5. FeatBag designates Feature Bagging.

**Table A3:** Best-performing outlier detection algorithms on all dataset configurations.

Dataset	LDF	Fixed-MiPo	Geometric	MiPo	IKAT
Podu Roș	ABOD	KNN	GMM	ABOD	IKAT
Podu Roș detour small	GMM	GMM	GMM	ABOD	IKAT
Podu Roș detour medium	GMM	GMM	ABOD	ABOD	IKAT
Podu Roș detour large	GMM	GMM	KNN	FeatBag	IKAT
Podu Roș route switch small	GMM	KNN	GMM	ABOD	IKAT
Podu Roș route switch med.	GMM	GMM	GMM	ABOD	IKAT
Podu Roș route switch large	GMM	GMM	GMM	ABOD	IKAT
Podu Roș point shift small	GMM	ABOD	KNN	ABOD	IKAT
Podu Roș point shift med.	GMM	GMM	KNN	ABOD	IKAT
Podu Roș point shift large	GMM	ABOD	KNN	ABOD	IKAT
Podu Roș shortcut small	GMM	KNN	GMM	ABOD	IKAT
Podu Roș shortcut medium	GMM	KNN	FeatBag	ABOD	IKAT
Podu Roș shortcut large	GMM	KNN	FeatBag	ABOD	IKAT
Podu Roș double loop small	GMM	GMM	KNN	ABOD	IKAT
Podu Roș double loop med.	GMM	GMM	KNN	ABOD	IKAT
Podu Roș double loop large	GMM	GMM	KNN	ABOD	IKAT
Porto AiSq5D	FeatBag	FeatBag	ABOD	FeatBag	IKAT
Porto AiSq5DP	ABOD	ABOD	ABOD	ABOD	IKAT
Porto AiSq5P	ABOD	ABOD	ABOD	ABOD	IKAT
Porto AiSq10D	ABOD	FeatBag	ABOD	ABOD	IKAT
Porto AiSq10DP	ABOD	ABOD	KNN	ABOD	IKAT
Porto AiSq10P	ABOD	INNE	KNN	ABOD	IKAT
Porto StSt5M	ABOD	KNN	ABOD	ABOD	IKAT
Porto StSt5R	ABOD	LSCP	ABOD	INNE	IKAT
Porto StSt10M	KNN	KNN	ABOD	LSCP	IKAT
Porto StSt10R	ABOD	KNN	FeatBag	ABOD	IKAT
Porto UnCh5M	FeatBag	FeatBag	FeatBag	LSCP	IKAT
Porto UnCh10M	KNN	LSCP	LSCP	KNN	IKAT
Hannover Trips	LOF	ABOD	ABOD	ABOD	IKAT
Edessa Trips	KNN	FeatBag	FeatBag	FeatBag	IKAT
